# Effect of the parasympathetic vasodilation on temperature regulation via trigeminal afferents in the orofacial area

**DOI:** 10.1186/s12576-020-00749-y

**Published:** 2020-03-31

**Authors:** Hanako Ohke, Toshiya Sato, Kohei Mito, Makoto Terumitsu, Hisayoshi Ishii

**Affiliations:** 1 Division of Dental Anesthesiology, Department of Human Biology and Pathophysiology, School of Dentistry, Health Sciences University of Hokkaido, Ishikari-Tobetsu, Hokkaido Japan; 2 Division of Physiology, Department of Oral Biology, School of Dentistry, Health Sciences University of Hokkaido, 1757 Kanazawa, Ishikari-Tobetsu, Hokkaido 061-0293 Japan

**Keywords:** Parasympathetic reflex vasodilation, Lingual nerve, Superior cervical sympathetic trunk, VIP, Skin temperature

## Abstract

The skin temperature (*T*_m_) of the orofacial area influences orofacial functions and is related to the blood flow (BF). Marked increases in BF mediated by parasympathetic vasodilation may be important for orofacial *T*_m_ regulation. Therefore, we examined the relationship between parasympathetic reflex vasodilation and orofacial *T*_m_ in anesthetized rats. Electrical stimulation of the central cut end of the lingual nerve (LN) elicited significant increases in BF and *T*_m_ in the lower lip. These increases were significantly reduced by hexamethonium, but not atropine. VIP agonist increased both BF and *T*_m_ in the lower lip. The activation of the superior cervical sympathetic trunk (CST) decreased BF and *T*_m_ in the lower lip; however, these decreases were significantly inhibited by LN stimulation. Our results suggest that parasympathetic vasodilation plays an important role in the maintaining the hemodynamics and *T*_m_ in the orofacial area, and that VIP may be involved in this response.

## Introduction

Local temperature (*T*_m_) in the orofacial area is generally considered to be important for the maintenance of orofacial functions such as oral sensations [1, 2] and wound healing [3]; abnormalities in *T*_m_ may be related to orofacial dysfunctions [4–6]. Blood flow (BF), regulated by the autonomic nervous system, in particular, the sympathetic vasoconstrictor fibers that secrete noradrenaline, plays a major role in the regulation of the *T*_m_ in the skin of the trunk and limbs [5, 7, 8]. However, the role of the autonomic nervous system in the regulation of *T*_m_ in the orofacial area remains unclear.

Two major vasomotor fibers consisting of parasympathetic vasodilator and sympathetic vasoconstrictor fibers are located in the orofacial area [9–11]. Parasympathetic vasodilator fibers have been demonstrated to originate from the pterygopalatine, otic, and submandibular ganglia in the orofacial area, and these fibers include acetylcholine and non-cholinergic neurotransmitters such as vasoactive intestinal polypeptide (VIP), as reported in physiological, pharmacological, and histochemical studies [12–15]. Previously, the activation of parasympathetic vasodilator fibers has been reported to occur through trigeminal afferent inputs leading to a rapid and marked increase in BF in orofacial tissues, such as lower lip [16], jaw muscles [17, 18] and salivary glands [15, 19]. On the other hand, sympathetic vasoconstriction is under tonic control from the superior cervical sympathetic trunk (CST) [20–22]. Therefore, parasympathetic vasodilation mediated through the trigeminal reflex mechanisms and interaction between parasympathetic and sympathetic fibers may play an important role in the regulation of both hemodynamics and *T*_m_ in the orofacial area. However, to the best our knowledge, the relationship between autonomic vasomotor responses and *T*_m_ in the orofacial tissues during trigeminal afferent input has not been evaluated so far.

**Fig. 1:**
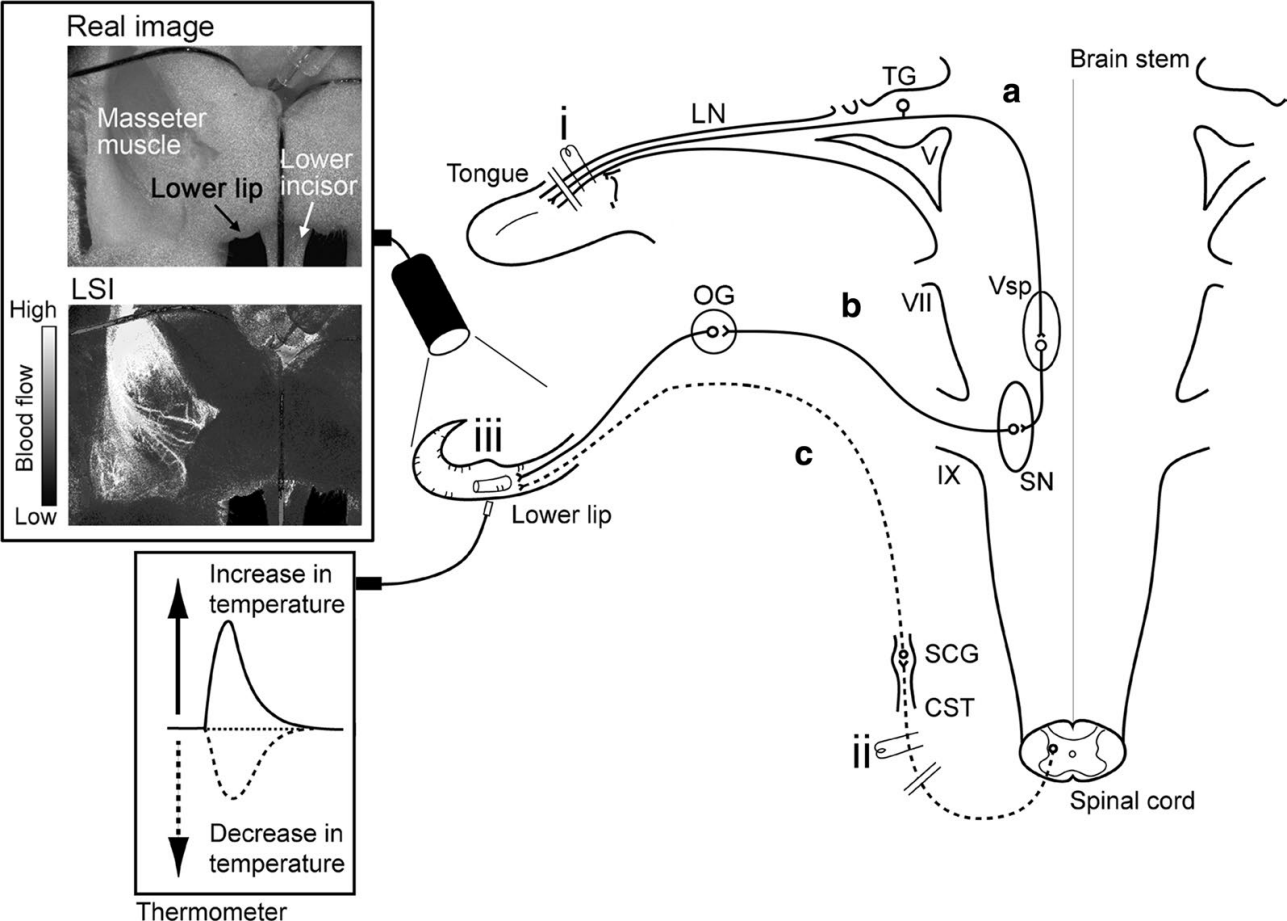
Schematic representation of the electrical stimulation sites and measurement sites of both local blood flow (BF) and temperature (*T*_m_) in rats. The stimulation sites were as follows: (i) central cut end of the lingual nerve (LN) and (ii) peripheral cut end of the superior cervical sympathetic trunk (CST). Local BF and *T*_m_ were measured in the (iii) lower lip (Real image), using laser speckle imaging (LSI) and a thermometer, respectively. The continuous lines indicate **a** trigeminal sensory inputs to the trigeminal spinal nucleus (V_sp_) in the brainstem and **b** parasympathetic vasodilator fiber to the lower lip from the salivatory nuclei (SN). The dashed lines indicate sympathetic vasoconstrictor fiber to the lower lip from the superior cervical ganglion (SCG) of the superior CST (**c**). *OG* otic ganglion, *TG* trigeminal ganglion, *V* trigeminal nerve root, *VII* facial nerve root, *IX* glossopharyngeal nerve root. Modified from Ishii et al. [17]

In the present study, we explored the effects of parasympathetic vasodilation evoked by the trigeminal-mediated reflex and sympathetic vasoconstriction (induced by the CST). In addition, the underlying mechanisms mediating these responses and their interactions on BF and *T*_m_ in the lower lip were examined using deeply urethane-anesthetized, artificially ventilated, vagotomized, and sympathectomized rats (Fig. 1).

## Methods

### Preparation of animals

The experiments were performed on 42 adult male Wistar rats (9–15 weeks of age, weighing 295–480 g). After induction of anesthesia using isoflurane, urethane (1 g/kg in a volume of 1 ml/100 g body weight) was injected subcutaneously into the backs of the animals. Room temperature was maintained at 25 ± 1 °C during the experiments. One femoral vein was cannulated to allow for the drug injection, and a femoral artery was cannulated and connected to a Statham pressure transducer to monitor the systemic arterial blood pressure (SABP) and heart rate (HR). The anesthetized animals were intubated, paralyzed by intravenous (iv) injection of pancuronium bromide (Mioblock; Organon, Teknika, the Netherlands; 0.6 mg/kg initially, supplemented with 0.4 mg/kg every hour or so after testing the level of anesthesia; see below), and artificially ventilated via a tracheal cannula with a mixture of 50% air and 50% O_2_. The ventilator (model SN-480-7; Shinano, Tokyo, Japan) was set to deliver a tidal volume of 8.5–10 cm^3^/kg at a rate of 20–23 breaths/min, and the end-tidal concentration of CO_2_ was determined by means of an infrared analyzer (Capnomac Ultima; Datex, Helsinki, Finland), as reported elsewhere [15, 17, 22]. This method of continuous ventilation has been shown to maintain the end-tidal concentration of CO_2_ at 40–45 mmHg. The changes in the end-tidal CO_2_ concentration following each treatment (from 45 to 35 mmHg) were independent of the changes in the BF and *T*_m_ measured by the present method (data not shown). Rectal temperature was maintained at 37–38 °C using a heating pad. Before the injection of additional pancuronium bromide, the adequacy of the depth of anesthesia was determined by the absence of the flexion response to a noxious stimulus, such as pinching the digit for approximately 2 s. The criterion for the maintenance of an adequate depth of anesthesia following paralysis was the absence of a reflex elevation of the SABP in response to a noxious stimulus. When the depth of anesthesia was considered inadequate, additional urethane (intermittent doses of 100 mg/kg, iv) was administered. At the end of the experiment, all rats were killed by an overdose (approximately 100 mg, iv) of pentobarbital sodium. The experimental protocols were reviewed and approved by the Animal Ethics and Research Committee and conducted in accordance with the Regulations for the Care and Use of Laboratory Animals of the Health Sciences University of Hokkaido (No. 075). All the animals were cared for in accordance with the recommendations in the current National Research Council guide.

### Measurement of cardiovascular parameters and local *T*_m_ in the orofacial area

Changes in BF in the lower lip (Fig. 1, iii) and skin of the dorsum of the foot on the left side were monitored using a laser speckle flowmeter (Omegazone; Omegawave, Tokyo, Japan), which obtains the high-resolution two-dimensional images in seconds, as described previously [15, 23, 24]. A 780-nm semiconductor laser was used to illuminate the surface of the orofacial area. The scattered light was filtered and detected by a charge-coupled device (CCD) camera positioned above the measuring sites. Raw speckle images (real images) corresponding to the number and velocity of moving red blood cells (BF) were collected by the CCD camera and transferred to a computer for analysis. Color-coded BF images (speckle images) were obtained in high-resolution mode (638 pixels × 480 pixels; 1 image/s). One BF image was generated by averaging the numbers obtained from 20 consecutive raw speckle images. The averaged signals in BF at the regions of interest (ROI), which indicated the highest increases in BF, were obtained using the pallet software installed in the Omegazone imaging system (Omegawave, Tokyo, Japan). The analog output of the equipment did not provide absolute values but demonstrated the relative changes in BF expressed in arbitrary units (a.u.) [25]. The SABP was recorded from a femoral catheter via a Statham pressure transducer. The HR, as well as the systolic, diastolic, and mean SABP were calculated from the SABP signals (*n* = 7, Table 1). Vascular conductance (VC) was calculated using the following equation:

$${\text{VC}}\;\left( {{\text{a}}.{\text{u}}./{\text{mmHg}}} \right) = {\text{BF}}\;\left( {{\text{a}}.{\text{u}}.} \right)/{\text{SABP}}\;({\text{mmHg}})$$

**Table 1:** Heart rate and systemic blood pressure responses associated each condition

Heart and blood pressure measurements	Baseline	LN simulation	CST simulation	CST + LN simulation
HR (beats/min)	422 ± 23	435 ± 22	426 ± 13	396 ± 7
Systolic SABP (mmHg)	121.9 ± 4.4	166.5 ± 8.6**	134.3 ± 8.2	178.4 ± 18.2*
Diastolic SABP (mmHg)	71.1 ± 6.1	108.2 ± 9.3**	84.1 ± 8.5	131.1 ± 16.8*
Mean SABP (mmHg)	88.1 ± 5.4	127.6 ± 8.9**	100.8 ± 8.4	146.9 ± 17.1*

Values in table are given as the mean ± standard error of the mean (SEM) (*n* = 7)

*LN* lingual nerve, *CST* cervical sympathetic trunk, *HR* heart rate, *SABP* systemic arterial blood pressure

Significant difference from baseline at *P < 0.05, **P < 0.001

**Fig. 2:**
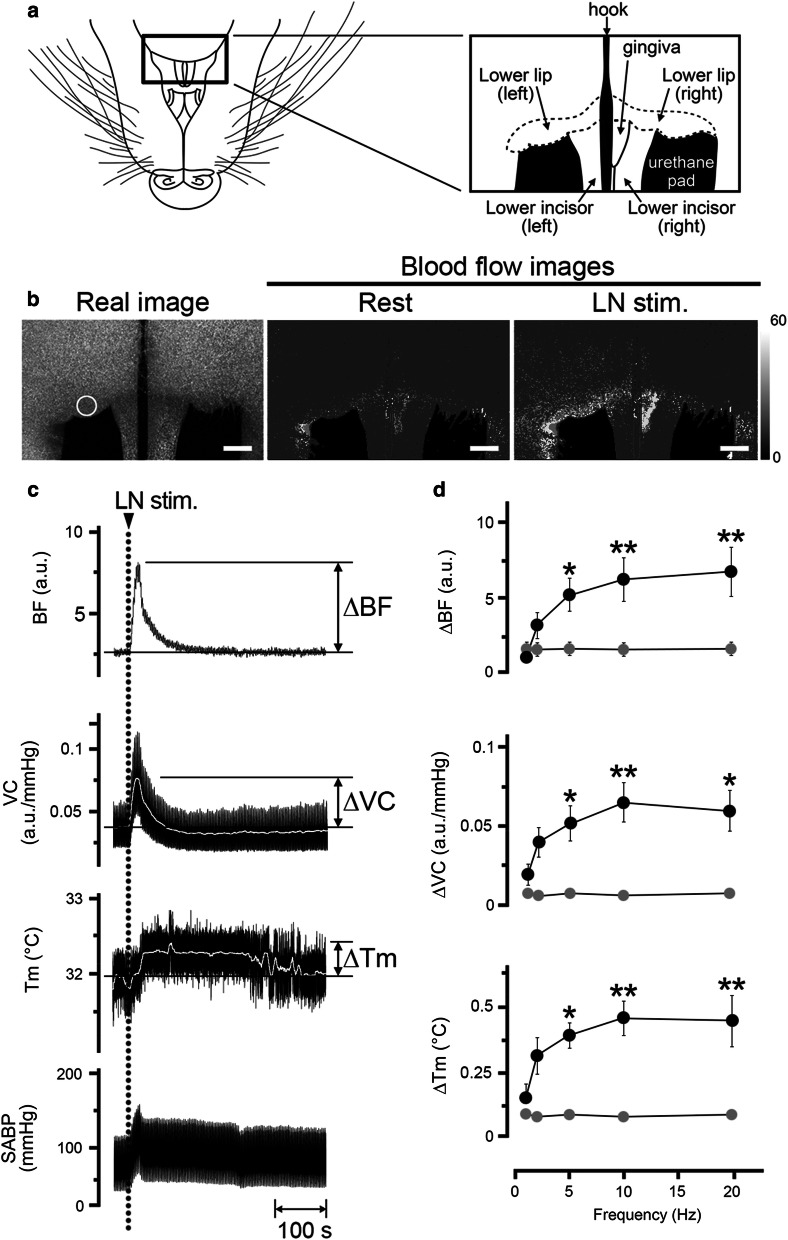
Relationships between hemodynamics and the *T*_m_ during trigeminal afferent inputs in the orofacial tissues in the rat. **a** Illustration of the area in the mandibular including lower lip in a supine rat. **b** Typical example of the real image and speckle images of the BF in the lower lip at the basal level (rest) and produced by left LN stimulation (LN stim.) for 20 s with a supramaximal voltage (20 V) at 20 Hz using 2-ms pulses. Scale bars represent 3.5 mm. **c** Changes in BF (a.u.; arbitrary units), vascular conductance (VC; a.u./mmHg) and *T*_m_ (°C) in the lower lip extracted from a region of interest (ROI) indicated by the white circles in **b**, and systemic arterial blood pressure (SABP; mmHg) evoked by LN stimulation (arrow head with dashed line). The white traces indicate the mean VC and *T*_m_ at the measuring site. **d** Mean ± standard error of the mean (SEM) of ∆BF, ∆VC, and ∆*T*_m_ in the lower lip (black symbols) and the skin of the dorsum of the foot (gray symbols) evoked by LN stimulation at 20 V and various frequencies (1–20 Hz; *n* = 6 in each group). The responses evoked by LN stimulation were determined by calculating the difference between the maximum value during the 10 min after stimulation and the baseline value. Statistical significance of the differences from the base value (at a frequency of 1 Hz) was assessed by ANOVA followed by a post-hoc test (Fisher’s PLSD). *P < 0.05, **P < 0.01 vs. base value

Local *T*_m_ was measured using a non-contact thermometer (PT-3S, OPTEX, Shiga, Japan) (Fig. 1), which measures the surface *T*_m_ of objects by caching the infrared energy emitted by the target objects (2.5 mm diameter). All data were collected online using a LabScribe2 data-acquisition system (iWorx systems, Washington, NH, USA). Changes (∆) in the parameters were assessed by measuring the heights of the maximum values from the baseline in the responses (Fig. 2) unless otherwise noted.

### Electrical stimulation of the LN and superior CST

The central cut end of the lingual nerve (LN; Fig. 1, i) and the peripheral cut end of the superior CST (Fig. 1, ii) were electrically stimulated using a bipolar silver electrode attached to an electrical stimulator (model SEN-7103; Nihon Kohden, Tokyo, Japan). For this purpose, the nerves were stimulated unilaterally under a binocular microscope. The LN was stimulated for 20 s using a supramaximal voltage (20 V) at various frequencies (1–20 Hz) for 2-ms pulse duration [15, 17, 24], either alone or in combination with CST stimulation (*n* = 6 in each group). Electrical stimulation of the CST was performed for periods of 2 min using a supramaximal voltage (10 V) and 2-ms pulse duration at various frequencies (0.5–5 Hz) [21]. These intensities in both the LN and CST have been reported to be optimal for inducing BF changes via each nerve stimulation as described in our previous studies [17, 21]. The period of LN stimulation chosen for the present study was 20 s because the parasympathetic vasodilator fibers were rapidly activated through the trigeminal-mediated reflex, as reported previously [17]. On the other hand, CST stimulation for 2 min appears to mimic the physiological forms of spontaneous tonic activity in the CST fibers supplying the orofacial vasculature. This is because vasoconstriction in the lower lip induced by CST stimulation reached stable levels within 1 min and sustained these levels during the stimulation (Figs. 5, 6) [21]. In all experiments, the cervical vagi and superior CST were bilaterally transected in the neck, before the stimulation to ensure that only non-vagal parasympathetic effects were examined.

### Pharmacological agents

All drugs were dissolved in sterile saline. The AGP-8633 (*n* = 6; 0.05–5 μg/ml, ANYGEN, Korea) was used as a VIP agonist. The following pharmacological interventions were performed: autonomic ganglion cholinergic blockade using hexamethonium bromide (*n* = 6; 10 mg/ml; Sigma-Aldrich, St. Louis, MO) and muscarinic cholinergic blockade using atropine sulfate (*n* = 6; 100 μg/ml; Mitsubishi Tanabe, Osaka, Japan). These drugs were perfused intravenously for 10 min at a flow rate of 0.1 ml/min using a syringe pump (Model ‘22’ Multisyringe; HARVARD, Holliston, MA). The administration of a similar volume of saline alone had no measurable effect on the cardiovascular parameters and local *T*_m_ (data not shown). The responses evoked by electrical stimulations after the administration of each drug were determined at least 10 min after injection because changes in BF and SABP reached a steady-state during this period. The magnitude of the response obtained following the administration of each blocking agent was expressed as a percentage of the control response recorded prior to its administration. The dose of hexamethonium chosen for the present study was 10 mg/ml; a similar dose markedly inhibited the increase in BF in the orofacial area, which was evoked by the activation of the parasympathetic vasodilator fibers through the trigeminal-mediated reflex [15, 17, 24]. The efficacy of the blockade using atropine was assessed by the absence of a vasodilator response in response to acetylcholine bromide (100 ng/kg, iv; Sigma-Aldrich, St. Louis, MO).

### Statistical analysis

All numerical data are presented as means ± standard error of the mean (SEM). The statistical significance of observed changes was assessed using paired Student’s *t* test or analysis of variance (ANOVA) followed by a post-hoc test [Fisher’s protected least significant difference (PLSD) test]. Differences in means were considered significant at *P* < 0.05. Data were analyzed using a Macintosh computer with StatView 5.0 (SAS Institute Inc., Cary, NC).

## Results

### Effects of electrical stimulation of the central cut end of the LN on the hemodynamics and local ***T***_m_ in the lower lip, and SABP

Figure 2 shows the changes in the BF, VC, and *T*_m_ of the lower lip, skin of the dorsum of the foot, and SABP before and after electrical stimulation of the central cut end of LN on the left side. The basal BF, VC, and *T*_m_ levels in the lower lip were 3.5 ± 0.3 a.u., 0.05 ± 0.01 a.u./mmHg, and 33.4 ± 0.6 °C, respectively. Electrical stimulation of the left LN for 20 s with 20 V and 2-ms pulses at 20 Hz increased BF, VC, and *T*_m_ in the lower lip on the left side, but not on the right side (Fig. 2b). Frequency–response curves were generated using stimulus trains (1–20 Hz) at 20 V (Fig. 2d). Significant changes in ∆BF, ∆VC, and ∆*T*_m_ evoked by LN stimulation in the lower lip occurred at frequencies above 5 Hz (for ∆BF, *F*_4, 25_ = 5.05, *P* < 0.01; for ∆VC, *F*_4, 25_ = 2.87, *P* < 0.05; for ∆*T*_m_, *F*_4, 25_ = 4.99, *P* < 0.01). In the contrast, electrical stimulation of the LN failed to affect the values in the skin of the dorsum of the foot (Fig. 2d). The animals exhibited normal systolic and diastolic pressures, mean SABP, and HR during rest (Table 1). The HR remained unchanged during LN stimulation (20 V, 20 Hz, 20 s) (*NS*, paired *t* test). However, significant differences in the SABP before and after LN stimulation were noted (*P* < 0.001, paired *t* test) (Table 1).

### Effects of pharmacological blocking agents on the increase in the BF and *T*_m_ in the lower lip evoked by LN stimulation

**Fig. 3:**
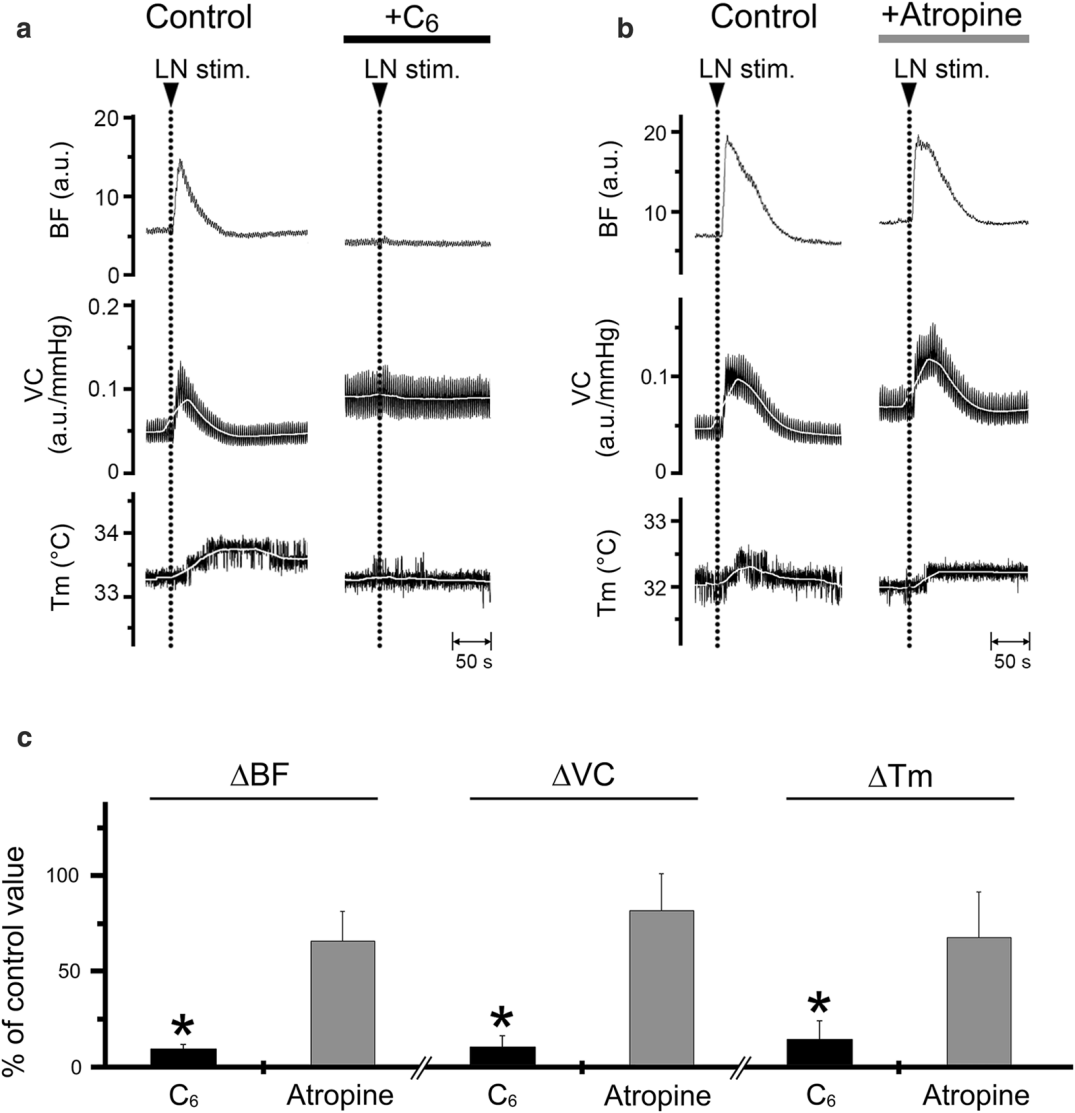
Effects of pharmacological blocking agents on the increase in BF and *T*_m_ evoked by LN stimulation in the lower lip. Typical examples of the effects of intravenous administration of hexamethonium (C_6_) at 10 mg/ml (**a**) and atropine (**b**) at 100 μg/ml for 10 min (0.1 ml/min) on changes in the BF, VC, and *T*_m_ in the lower lip on the left side evoked by left LN stimulation (20 s, 10 V, 20 Hz, 2-ms). **c** Mean ± SEM of changes in the ∆BF, ∆VC, and ∆*T*_m_ in the lower lip evoked by LN stimulation with administration of C_6_ (black bars) and atropine (gray bars; *n* = 6 in each group). The responses evoked by LN stimulation with C_6_ and atropine were determined by calculating the differences between the maximum values during 10 min after stimulation with C_6_ and atropine and the baseline values. Each value is expressed as a percentage of the response before treatment (control). Statistical significance was assessed by ANOVA followed by a post-hoc test (Fisher’s PLSD). *P < 0.001 vs. control

Increases in BF, VC, and *T*_m_ in the lower lip on the left side evoked by left LN stimulation (20 s, 20 V, 20 Hz, 2-ms) were almost abolished by the intravenous administration of hexamethonium (C_6_, 10 mg/ml) (Fig. 3a). Significant differences in the ∆BF, ∆VC, and ∆*T*_m_ evoked by LN stimulation in the lower lip before and after hexamethonium administration were observed (for ∆BF, *F*_2, 15_ = 47.5, *P* < 0.001; for ∆VC, *F*_2, 15_ = 19.6, *P* < 0.001; for ∆*T*_m_, *F*_2, 15_ = 24.9, *P* < 0.001, ANOVA followed by Fisher’s PLSD; Fig. 3c). The responses returned close to the initial value at 30–60 min after hexamethonium administration (data not shown). Administration of atropine (100 μg/ml) had no effect on the response (Fig. 3b, c). The HR values at 10 min after the administration of hexamethonium and atropine were 371 ± 19 and 400 ± 5 beats/min, respectively, and mean SABP values 10 min after the administration of hexamethonium and atropine were 60.5 ± 2.9 mmHg and 108.7 ± 24.6 mmHg, respectively. Statistically significant differences in the mean SABP before and after the administration of hexamethonium (*P* < 0.001), but not atropine, were noted.

### Effects of exogenously applied VIP agonist on the hemodynamics and *T*_m_ in the lower lip

**Fig. 4:**
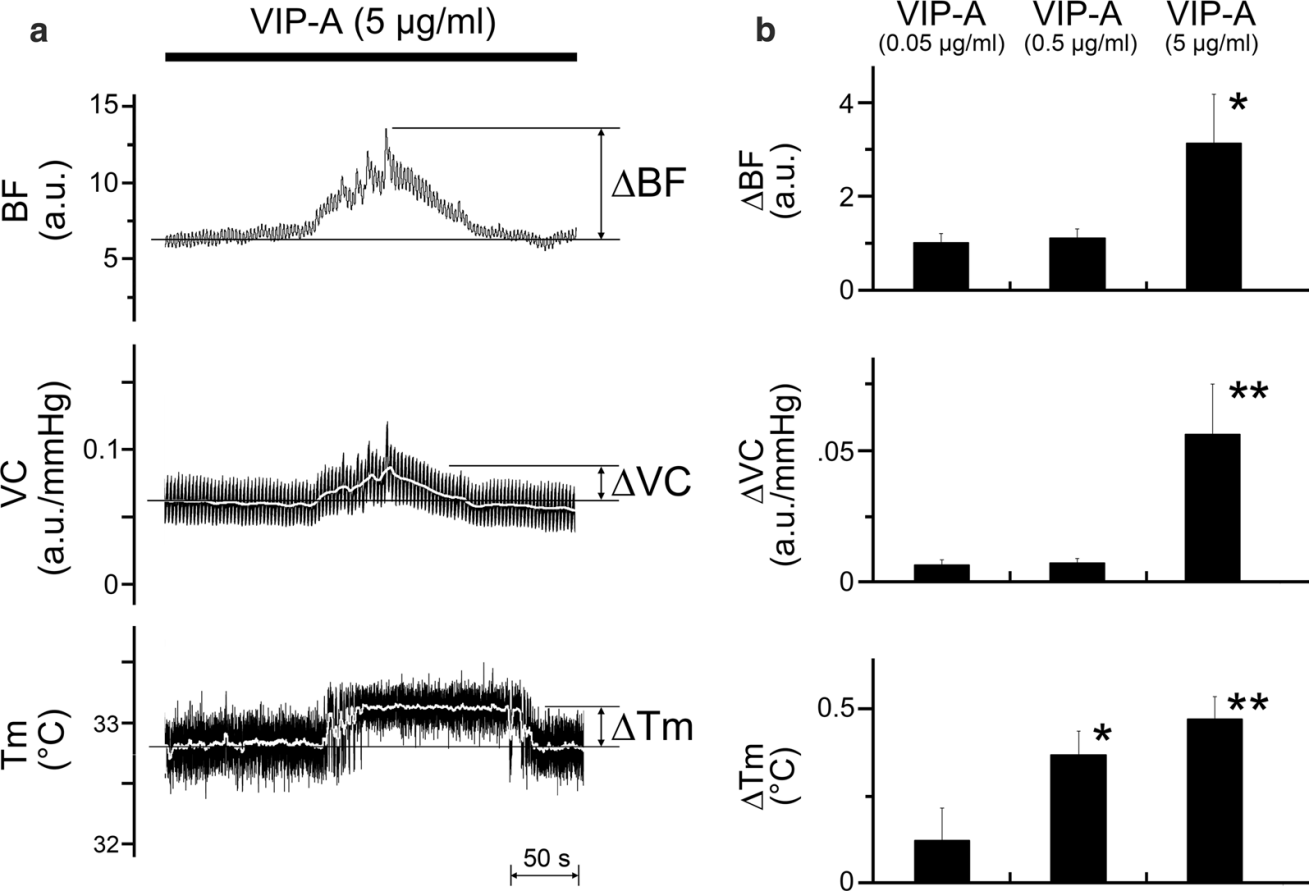
Effects of an exogenously applied VIP agonist on the hemodynamics and *T*_m_ in the lower lip. **a** Typical examples of the effects of intravenous administration of VIP agonist (VIP-A) at 5 μg/ml for 10 min (0.1 ml/min) on changes in BF, VC, and *T*_m_ in the lower lip on the left side. **b** Mean ± SEM of changes in the ∆BF, ∆VC, and ∆*T*_m_ in the lower lip evoked by the drug at 0.05–5 μg/ml (*n* = 6 in each group). The responses evoked by the administration of a VIP agonist were determined by calculating the difference between the maximum value during the 10 min after its administration and the value at baseline. *P < 0.05, **P < 0.01 vs. base value

Figure 4a shows the effects of intravenous administration of a VIP agonist at (5 μg/ml) on the BF, VC, and *T*_m_ in the lower lip on the left side. The administration of the VIP agonist induced increases in BF, VC, and *T*_m_ in the lower lip in a dose-dependent manner (0.05–5 μg/ml; Fig. 4b). Significant changes in ∆BF and ∆VC were evoked by the agonist at 5 μg/ml; likewise, the changes in ∆*T*_m_ were evoked by the agonist at dose above 0.5 μg/ml (for ∆BF, *F*_2, 15_ = 5.78, *P* < 0.05; for ∆VC, *F*_2, 15_ = 8.59, *P* < 0.01; for ∆*T*_m_, *F*_2, 15_ = 7.14, *P* < 0.01; Fig. 4b). The HR at 10 min after each dose of VIP was 410 ± 17 beats/min. No statistically significant differences in the HR before and after its administration were observed. The mean SABP 10 min after VIP agonist administration at dose of 0.05, 0.5, and 5 μg/ml were 113.7 ± 5.2, 111.9 ± 5.1, and 76.1 ± 5.2 mmHg, respectively. A significant difference in mean SABP before and after the administration of VIP agonist at 5 μg/ml was observed (*P* < 0.001, paired *t* test).

### Effects of electrical stimulation of the peripheral cut end of the CST on the hemodynamics and *T*_m_ in the lower lip, and the SABP

**Fig. 5:**
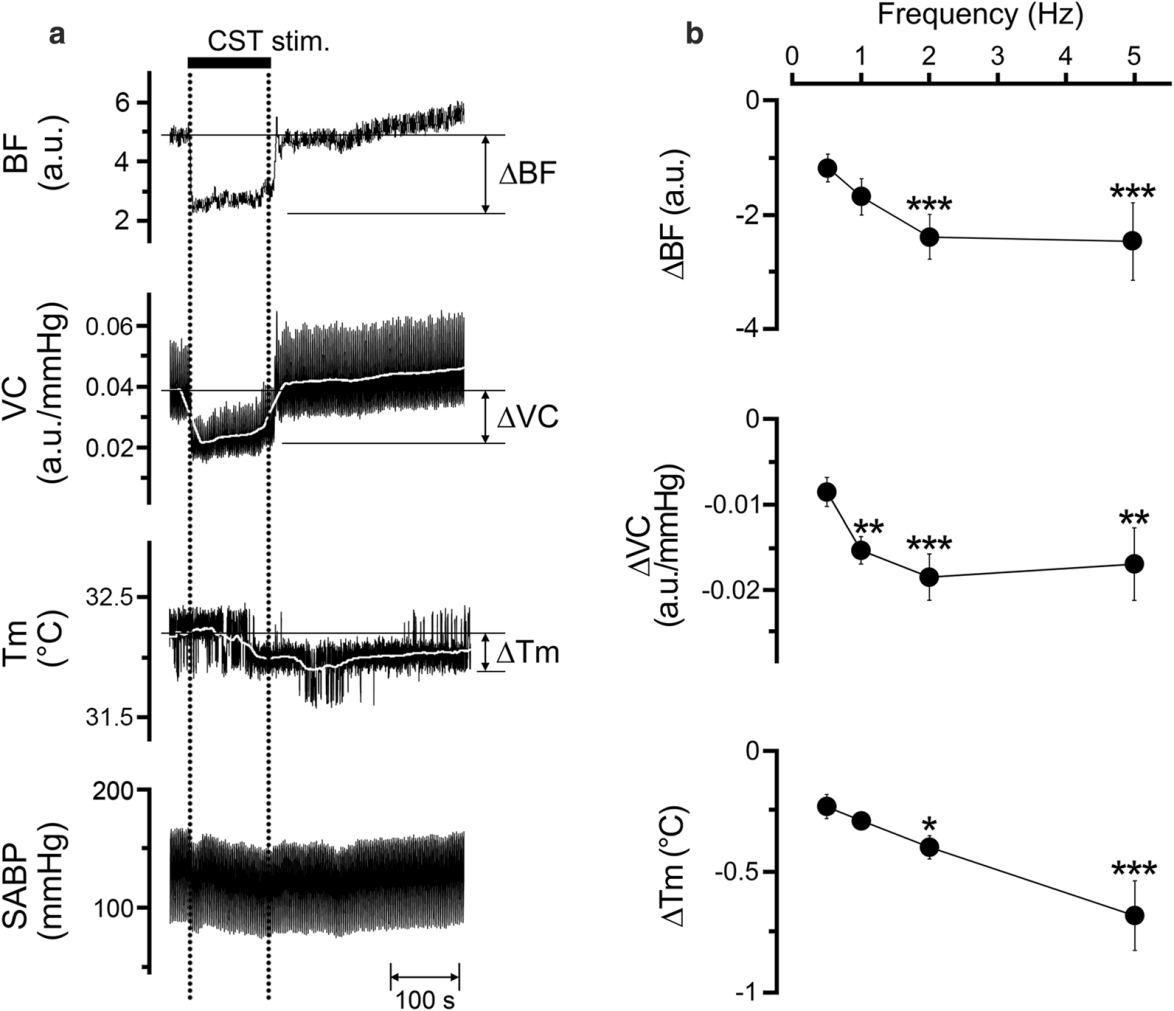
Sympathetic effects on the hemodynamics and *T*_m_ in the lower lip, and SABP. **a** Typical examples of changes in BF, VC, and *T*_m_ in the lower lip on the left side, extracted from the ROI indicated by the white circles in Fig. 2b, and SABP evoked by electrical stimulation of the peripheral cut end of the left CST (CST stim.; horizontal bar with dashed lines) for 2 min with a supramaximal voltage (10 V) at 5 Hz using 2-ms pulses. **b** Mean ± SEM of ∆BF, ∆VC, and ∆*T*_m_ in the lower lip (black symbols) evoked by CST stimulation at 10 V and various frequencies (0.5–5 Hz; *n* = 6 in each group). The responses evoked by CST stimulation were determined by calculating the difference between the maximum value during the 10 min after stimulation and the baseline value. *P < 0.05, **P < 0.01, ***P < 0.001 vs. base value

Figure 5 shows the changes in the BF, VC, and *T*_m_ in the lower lip on the left side, and in SABP before and after electrical stimulation of the peripheral cut end of the left CST. Electrical stimulation for 2 min with 10 V at 5 Hz using 2-ms pulses of the left CST decreased BF, VC, and *T*_m_ in the lower lip (Fig. 5a). Frequency–response curves were generated using stimulus trains (0.5–5 Hz) at 10 V (Fig. 5b). Significant changes in ∆BF, ∆*T*_m_, and ∆VC evoked by CST stimulation in the lower lip occurred at frequencies above 1 and 2 Hz (for ∆BF, *F*_3, 20_ = 3.18, *P* < 0.05; for ∆VC, *F*_3, 20_ = 3.39, *P* < 0.05; for ∆*T*_m_, *F*_3, 20_ = 6.21, *P* < 0.01). The HR and SABP remained unchanged during CST stimulation (Table 1). No significant differences in HR and mean SABP were observed before and after CST stimulation.

### Effects of CST stimulation in combination with LN stimulation on the hemodynamics and *T*_m_ in the lower lip, and SABP

**Fig. 6:**
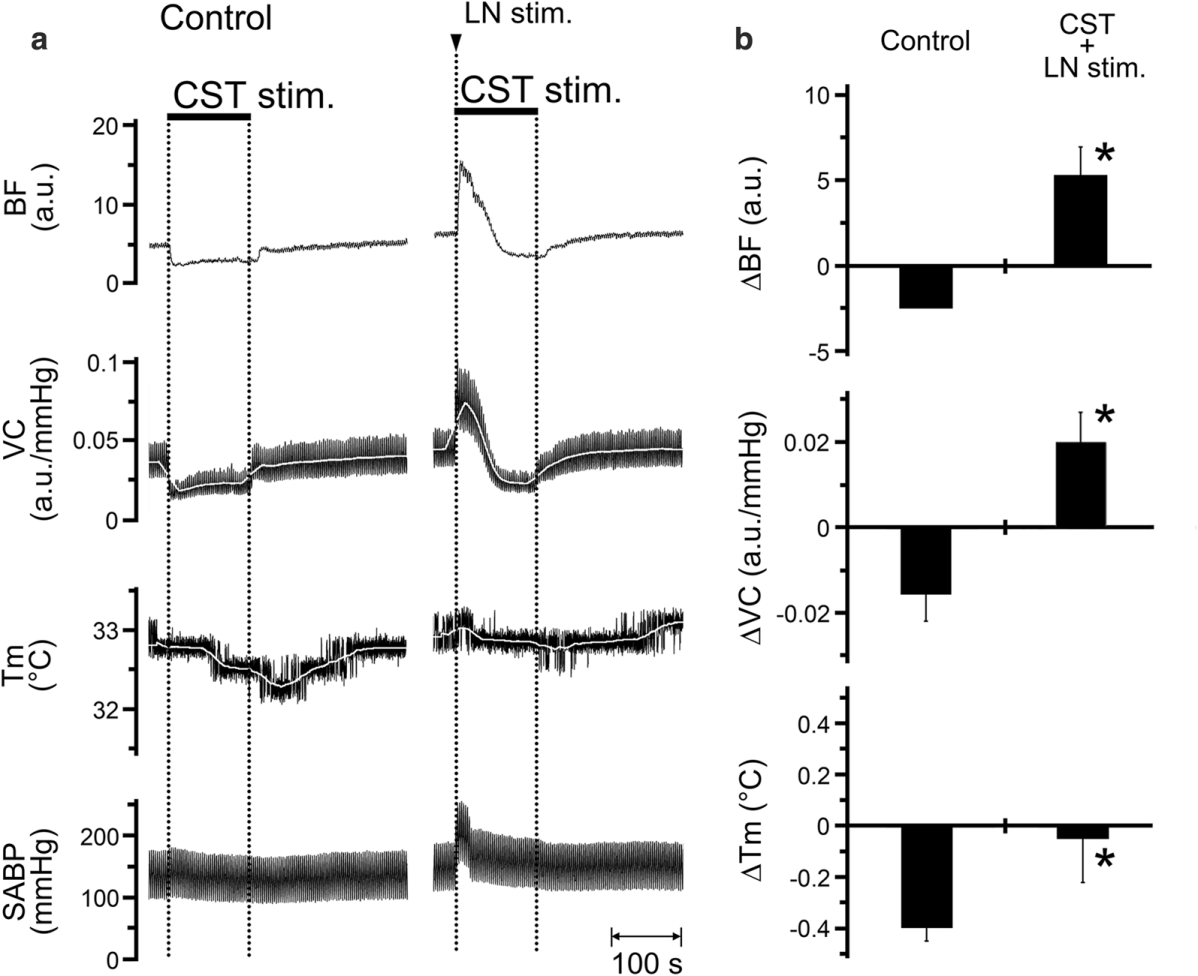
Interactions between trigeminally mediated parasympathetic vasodilation and sympathetic vasoconstriction in the regulation of the hemodynamics and *T*_m_ in the orofacial area. **a** Typical examples of the changes in BF, VC, and *T*_m_ in the lower lip on the left side, and SABP evoked by left CST stimulation (CST stim.) alone (2 min, 10 V, 5 Hz, 2-ms; control) and in combination with left LN stimulation (20 s, 20 V, 20 Hz, 2-ms). Changes (∆) in the parameters were assessed by calculating the sum of the maximum ( +) and minimum (−) values from the baseline in the responses. **b** Mean data ± SEM of ∆BF, ∆VC, and ∆*T*_m_ in the lower lip (black bars) evoked by left CST stimulation alone (control) and in combination with LN stimulation (CST + LN stim.; *n* = 6 in each group). Statistical significance of the differences from control was assessed by paired Student’s *t* test. *P < 0.001 vs. control

Figure 6 shows the effects of CST stimulation (2 min, 10 V, 5 Hz, 2-ms) alone (control) and in combination with LN stimulation (20 s, 20 V, 20 Hz, 2-ms) on the left side. Decreases in BF, VC, and *T*_m_ in the lower lip evoked by CST stimulation were inhibited by LN stimulation (Fig. 6a). Furthermore, significant differences in ∆BF (*P* < 0.01), ∆VC (*P* < 0.001), and ∆*T*_m_ (*P* < 0.05) in the lower lip were observed between CST stimulation alone and in combination with LN stimulation (paired *t* test; Fig. 6b). HR remained unchanged during each stimulus condition (Table 1). Significant differences in SABP before and after CST stimulation in combination with LN stimulation were observed (*P* < 0.05, paired *t* test; Table 1).

## Discussion

Our results showed that electrical stimulation of the central cut end of the LN in cervically sympathectomized and vagotomized rats significantly increased the BF and *T*_m_ in a frequency-dependent manner on the ipsilateral side of the lower lip (Fig. 2). The observed increases in BF and *T*_m_ were found to be unrelated to changes in the SABP due to the following reasons: the VC in each site was significantly increased by LN stimulation; no significant increases in BF and *T*_m_ were noted on the contralateral side of the lower lip and the skin of the dorsum of the foot (measured simultaneously; Fig. 2); and no significant differences in the HR before and after LN stimulation were observed (Table 1). These results suggest that increases in BF and Tm elicited by LN stimulation are not a passive result of any evoked SABP or HR changes and that these increases are likely the result of vasodilation. The evocation of vasodilation through the trigeminal afferent may play an important role in the regulation of the hemodynamics and *T*_m_ in the orofacial area under physiological conditions. This is because LN stimulation-induced BF increase has been reported in some orofacial tissues including the lower lip, regardless of the presence (CST intact) or absence (sectioning of the CST) of the sympathetic innervation [17].

The increase in both BF and *T*_m_ in the lower lip followed by LN stimulation was prominently reduced by the intravenous administration of hexamethonium, whereas the administration of atropine had no significant effect on either response (Fig. 3). These results suggest that LN stimulation-induced increases in BF and *T*_m_ in the lower lip are mediated by the activation of parasympathetic reflex vasodilation, which is mediated by final neurons via a non-cholinergic response. This is in accord with the observations that LN stimulation-induced BF increase in the rat lower lip is almost mediated through the atropine-resistant parasympathetic vasodilation [17]. The neural mechanisms underlying non-cholinergic parasympathetic vasodilation in the orofacial area are not fully understood. However, this response may be mediated, in part, by VIP because the intravenous administration of a VIP agonist induced an increase in both BF and *T*_m_ in the lower lip in a dose-dependent manner (Fig. 4). This was supported by the findings that VIP immunoreactivity is observed in the otic and submandibular ganglion and in the nerve fibers that innervate the blood vessels in the lip [12, 26]. Furthermore, the intravenous administration of VIP also induces vasodilation in the masseter muscle [14] and submandibular gland [15], which is markedly suppressed by a selective VIP receptor antagonist. However, further investigations are necessary to establish the neural mechanisms underlying the non-cholinergic parasympathetic vasodilation in the orofacial area.

The increase in BF in the lower lip evoked by LN stimulation was phasic, whereas the increase in *T*_m_ appeared to be a longer response. The precise reasons for the differences in the durations of the responses are unclear, but it may be due to the widely induced increase in BF during LN stimulation in the lower lip (Fig. 2). This suggests that parasympathetic reflex vasodilation may be involved in maintaining a continuous *T*_m_ in the orofacial area.

Electrical stimulation of the peripheral cut end of the CST significantly decreased BF and *T*_m_ in a frequency-dependent manner in the lower lip, on the ipsilateral side (Fig. 5). The decrease induced by CST stimulation appeared to be vasoconstriction because no significant changes in the SABP and HR were observed during its stimulation (Table 1). This indicates that sympathetic vasoconstriction evoked by excess sympathetic activity reduces BF and *T*_m_ in the orofacial tissues. Furthermore, the simultaneous stimulation of the CST and LN increased BF and VC; however, *T*_m_ was not increased in the lower lip (Fig. 6). These results suggest that the hemodynamics and *T*_m_ in the orofacial area could be susceptible to sympathetic activity. Modulation of the sympathetic nerve activity associated with stress and chronic pain, such as fibromyalgia, is known to induce changes in cardiovascular parameters, such as blood pressure and regional BF [27–29]. In addition, both vasoconstriction and decreased *T*_m_ during sympathoexcitation are thought to be associated with fibromyalgia in the tender points above the skin [30]. These observations suggest that disturbances in BF and *T*_m_ may play a role in the development of orofacial dysfunctions relevant to autonomic abnormalities.

Decreases in BF and *T*_m_ in the lower lip evoked by CST stimulation were inhibited significantly by LN stimulation (Fig. 6). This result indicates that the parasympathetic reflex vasodilation evoked by trigeminal afferent inputs compensates for the hypoperfusion of the BF, which induces a decrease in *T*_m_ in the orofacial area. Elevated thermal conditions are thought to be involved in the functional properties of the orofacial tissues. Wound repair of the orofacial areas, such as the oral mucosa, has been reported to be faster than that in the dorsal skin [31, 32]; the thermosensitive transient receptor potential vanilloid 3 (TRPV3) is known to contribute to rapid wound healing in the oral epithelia [3]. Furthermore, the decreased sensitivity of the orofacial tissues (such as orofacial skin in lower lip and tongue tip) to hot and cold may be due to the high baseline *T*_m_ in the orofacial area [1, 33]. These observations suggest that the trigeminal–parasympathetic reflex vasodilation for the maintenance of *T*_m_ may be involved in the functions of the oral epithelium and the sensory systems in the orofacial area under extensive stimuli during mastication, swallowing and speech.

## Conclusion

In conclusion, our results suggest that parasympathetic vasodilation plays an important role in the maintaining the hemodynamics and *T*_m_ in the orofacial area, and that VIP may be involved in this response. The increase in BF evoked by parasympathetic reflex vasodilation, which induces an increase in the local *T*_m_, may be important for the compensation of hypoperfusion and for the decrease in *T*_m_, which are mediated by the CST. Further studies on the precise neural mechanisms, including molecular properties of the trigeminal–parasympathetic reflex vasodilation and the relationships between *T*_m_ and orofacial functions, will provide a better understanding of the functional properties with regard to the autonomic vasomotor responses in the orofacial area; in addition, the etiology of orofacial disorders, such as chronic pain and fibromyalgia related to the disturbances of the autonomic nervous system, may be further elucidated.

## Data Availability

All relevant data are within the paper.

## Abbreviations

ANOVA: Analysis of variance
a.u.: Arbitrary units
BF: Blood flow
CCD: Charge-coupled device
CST: Superior cervical sympathetic trunk
C_6_: Hexamethonium
Fisher’s PLSD: Fisher’s protected least significant difference test
HR: Heart rate
iv: Intravenous
IX: Glossopharyngeal nerve root
LN: Lingual nerve
LSI: Laser speckle imaging
OG: Otic ganglion
ROI: Region of interest
SABP: Systemic arterial blood pressure
SCG: Superior cervical ganglion
SEM: Standard error of the mean
SN: Salivatory nuclei
*T*_m_: Local temperature
TG: Trigeminal ganglion
V: Trigeminal nerve root
VII: Facial nerve root
VIP: Vasoactive intestinal polypeptide
Vsp: Trigeminal spinal nucleus
